# The Effect of Sodium Dodecyl Sulphate Additives on the Electrochemical Performance of Aqueous Zinc Ion Batteries

**DOI:** 10.3390/molecules30030529

**Published:** 2025-01-24

**Authors:** Na Chen, Ying Huang, Yuan Lv, Wenju Wang

**Affiliations:** 1 Yinchuan Power Supply Company, State Grid Ningxia Electric Power Co., Ltd., Yinchuan 750011, China; 2 School of Energy and Power Engineering, Nanjing University of Science and Technology, Nanjing 210094, China; 3 Energy Technology Institute, Nanjing 210094, China

**Keywords:** zinc ion battery, zinc anode, electrolyte, zinc dendrite, interfaces, additives

## Abstract

Aqueous zinc ion batteries are considered one of the most promising energy storage devices due to their high safety, low cost, and ease of fabrication. However, the growth of anode dendrites and continuous side reactions during cycling limit the practical application of zinc ion batteries. In this paper, sodium dodecyl sulfate (SDS) was used as an aqueous electrolyte additive to improve the surface deposition of Zn^2+^. The experimental results show that the SDS electrolyte additive forms a protective layer on the anode surface through electrostatic action and inhibits the growth of dendritic protruding dendrites by increasing the zinc deposition overpotential, as well as by limiting the two-dimensional diffusion of Zn^2+^ on the negative electrode surface of the aqueous zinc ion battery. As a result, adding SDS improves the discharge specific capacity of NVP/Zn batteries at high voltages and results in improved capacity retention. The cycling stability of NVP/Zn batteries was greatly enhanced by using a battery containing 1% SDS that still had a discharge specific capacity of 71 mAh/g after 100 cycles at a charging current density of 1 C, with a capacity retention rate of 89%. This work provides a simple and feasible solution to the anode problem of aqueous zinc ion batteries.

## 1. Introduction

In recent years, green and environmentally friendly energy sources such as solar, wind, tidal, and geothermal energy have been widely used in human life. Nevertheless, the utilization of these energy sources is limited by nature, geographical conditions, and spatial distribution, and there is an urgent need to develop energy storage devices with features such as high safety and low cost. Aqueous zinc ion batteries are expected to be used in areas such as electric two-wheelers, small home energy storage systems, and power system energy storage because of their high safety, low cost, ease of manufacturing, and green features [1]. Notably, the zinc anode has a high theoretical capacity (820 mAh/g) and a low redox potential of −0.76 V vs. the Standard Hydrogen Electrode (SHE) [2,3]. However, the problems of dendrite growth and side reactions in conventional aqueous zinc ion batteries seriously affect the reversibility of the Zn anode and greatly reduce the cycle life of the battery [4,5,6,7]. The uncontrolled generation of dendrites may penetrate the diaphragm and cause a short circuit in the zinc ion battery, and once the dendrites leave the metal surface of the negative electrode, “dead zinc” will be generated. It is difficult to produce aqueous zinc ion batteries to isolate the electrolyte from the negative electrode solid electrolyte (SEI), and a hydrogen emission reaction (HER) occurs when zinc is deposited [8]. Hydrogen precipitation may lead to localized OH^−^ enrichment, producing a large number of by-products [9]. In zinc ion batteries, how to inhibit the generation of dendrites and prevent by-products is a top priority.

To solve these problems, researchers have put much effort into varying solutions, including layered anode structure design, surface modification [10,11], the construction of artificial interfaces [12,13], and electrolyte composition optimization [14]. Among these methods, doping additives to modulate the electrolyte composition of aqueous zinc ion batteries is an effective way to control the atmosphere of Zn^2+^ coordination, which contributes to the reversible deposition/stripping of zinc [15]. Huang et al. used ethyl ether (Et_2_O) as an additive for aqueous zinc ion batteries and found that Et_2_O molecules were preferentially adsorbed on the tip of the zinc anode surface of aqueous zinc ion batteries, which prevented the preferential deposition of the cation Zn^2+^ in the electrolyte on the tip of the anode surface of the aqueous zinc ion batteries and improved the process of zinc deposition on the anode zinc metal surface of the aqueous zinc ion batteries [16,17]. Liu et al. added Hexadecyl trimethyl ammonium Bromide (CTAB) to a zincate electrolyte, which reduced the initial potential of zinc deposition, decreased the growth rate of dendrites, and promoted denser and more uniform zinc deposition [18]. Li et al. found that polyacrylamide (PAM) additives could make the negative electrode surface of aqueous zinc ion batteries’ zinc deposition more uniform with the help of the strong adsorption of acyl groups, and the 2D diffusion of Zn^2+^ was more rapid and did not aggregate into dendrites [19]. Hou et al. added a sodium dodecyl sulfate (SDS) surfactant to an electrolyte to extend the electrochemical stability window of aqueous electrolytes to 2.5 V. They found that SDS molecules could be adsorbed on the electrode surface by electrostatic adsorption, thus effectively inhibiting the evolution of hydrogen or oxygen [20]. Their results suggest that to achieve the uniform deposition of negative electrode zinc metal in aqueous zinc ion batteries, it is necessary to screen those ions with lower reduction potentials than Zn^2+^ to be added to the electrolyte. The cations of the additives generally accumulate around the initially growing zinc “tip” on the zinc metal surface, forming an electrostatic shield, and this effect repels the later Zn^2+^, which can only surround the “tip”, and these cations cannot be electrochemically deposited at this potential.

Herein, we propose a simple and feasible strategy to promote the uniform deposition of zinc ions in aqueous batteries through the use of surfactant additives. More specifically, sodium dodecyl sulfate (SDS), as an anionic surfactant, tends to adsorb on the surface of the zinc anode to form a hydrophobic interface to ensure the uniform deposition of zinc ions. Unlike conventional surfactants, the introduction of SDS does not affect the solvent structure. Due to the natural hydrophobicity of SDS, the ligand zinc ions with high site resistance in aqueous solutions will not aggregate at the preferential nucleation sites, thus suppressing undesirable phenomena such as surface corrosion, hydrogen precipitation, and dendrite growth without sacrificing the deposition kinetics of the zinc ions, and improving the energy density and cycle stability of the battery. Thanks to these advantages of SDS, the Na_3_V_2_(PO_4_)_3_/Zn battery (NVP/Zn battery) with 1% SDS electrolyte still has a discharge specific capacity of 71 mAh/g after 100 cycles at 1 C current density after charging, the capacity retention rate is as high as 89%, and the cycling stability of the NVP/Zn battery is greatly enhanced. In addition, SDS acts as a buffer to effectively enhance the chemical stability of the Zn anode and slow down its corrosion process. This strategy provides a new way to develop advanced electrolytes for application in aqueous energy storage systems.

## 2. Results and Discussion

**Figure 1:**
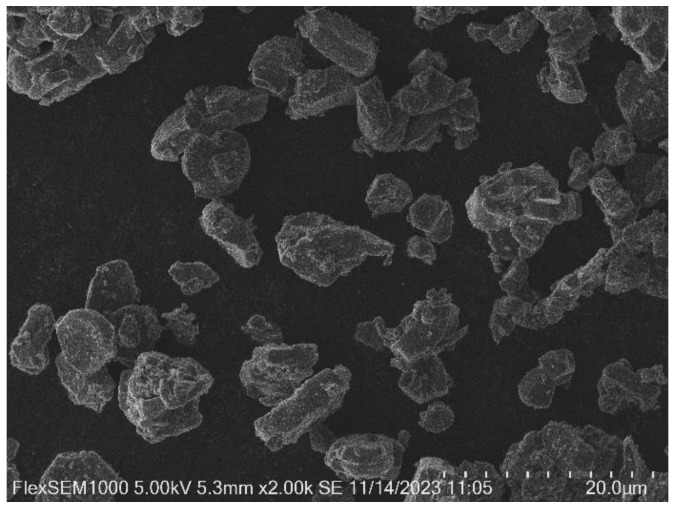
SEM image of sodium vanadium phosphate NVP.

To investigate the changes in the electrode materials during the cycling process, the positive electrode material Na_3_V_2_(PO_4_)_3_ was characterized by SEM after 100 cycles, and the results are shown in Figure 1. It can be seen that cracks appear on the surface of NVP in a large area and the surface is rough and uneven; it is observed under high magnification that the surface structure becomes loose and the morphology is more fluffy, mostly flocculent, and its morphology is mostly granular with a relatively compact structure.

**Figure 2:**
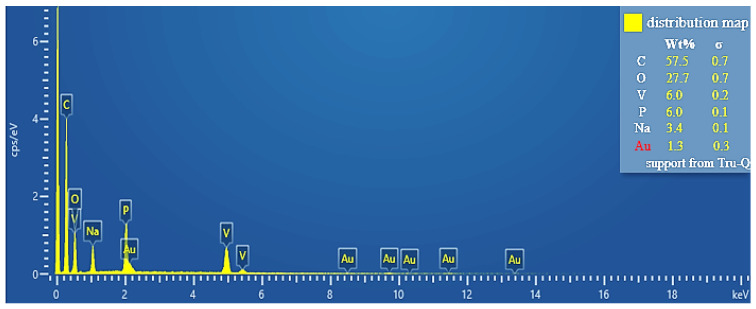
EDS diagram for NVP.

**Figure 3:**
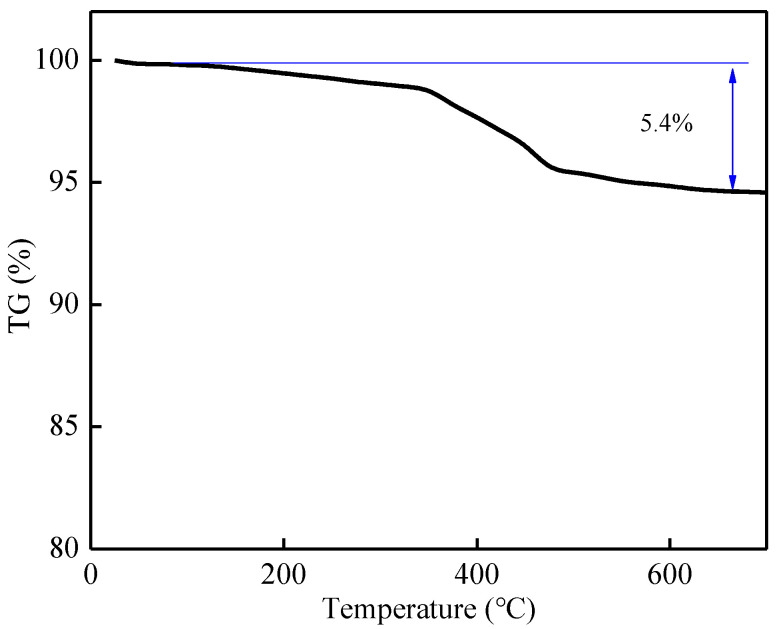
TG chart for NVP.

The elemental composition of NVP was observed by an EDS test, and the results are shown in Figure 2. It can be seen that the elemental ratio of NVP is close to Na_3_V_2_(PO_4_)_3_, and through the elemental ratio analysis, combined with the thermogravimetric analysis (see Figure 3), it is found that the residual carbon coating content of the resulting NVP powder is 5.4 wt%, the sodium vanadium phosphate prepared in this paper contains an obvious carbon layer, and the synergistic effect of carbon and NVP improves the electrical conductivity of the material and the ability of high-current charge/discharge. Thanks to the open Sodium Super Ion Conductor (NASICON) framework and the composite of a three-dimensional conductive carbon network, the composites have a more excellent ion diffusion rate and electron migration rate.

The lattice constants of Na_3_V_2_(PO_4_)_3_ are a = 8.895 Å, b = 8.889 Å, and c = 21.764 Å. The lattice constants of NaV_2_(PO_4_)_3_ are a = 8.5842 Å, b = 8.5843 Å, and c = 21.6034 Å. The crystal lattice constants are a = 8.5842 Å, b = 8.5843 Å, and c = 21.6034 Å. The lattice constants are a = 8.895 Å, b = 8.889 Å, and c = 21.764 Å. Compared with Na_3_V_2_(PO_4_)_3_, the a-axis and b-axis of NaV_2_(PO_4_)_3_ contracted and the c-axis did not change much after disodium removal, and the crystal skeleton structure did not change significantly, indicating that Na_3_V_2_(PO_4_)_3_ has excellent structural stability as a battery cathode material during charging and discharging.

It is worth noting that the ion de-embedding platform is an important parameter for sodium vanadium phosphate electrode materials that can be used in aqueous batteries. Although the presence of unstable divalent vanadium makes it difficult to directly synthesize Na_4_V_2_(PO_4_)_3_ with all the Na^+^ sites occupied, it can be obtained by electrochemical reactions at voltages less than 2 V. The presence of unstable divalent vanadium makes it difficult to directly synthesize Na_4_V_2_(PO_4_)_3_ with all the Na^+^ sites occupied. Therefore, Na_3_V_2_(PO_4_)_3_ can be directly used as a positive electrode material by the cyclic process of sodium removal and then sodium embedding, and can be used as a negative electrode material by the cyclic reaction of sodium embedding and then sodium removal.

**Figure 4:**
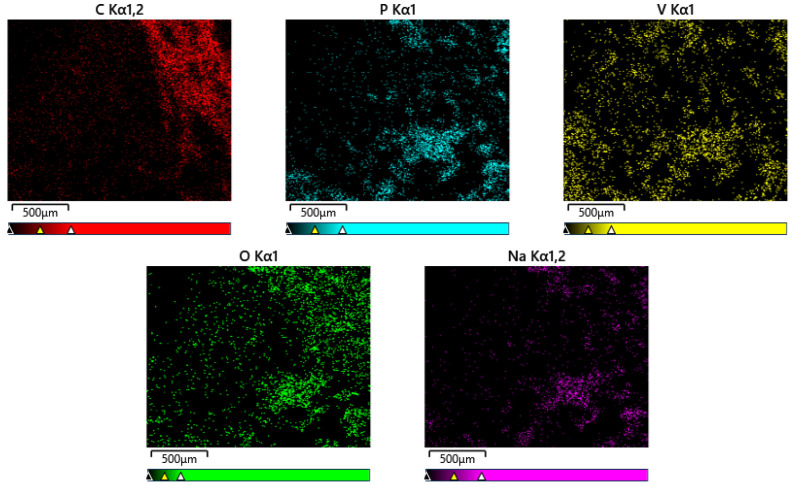
Distribution of layered elements of NVP.

The element distribution on the surface of NVP is observed through the element distribution diagram, and the results are shown in Figure 4. It can be seen that the elemental distribution on the surface of NVP is relatively uniform, and the overall generated NVP has many active sites, which is conducive to the role of the electrolyte, and it can effectively reduce the diffusion path of Zn^2+^ and accelerate the diffusion rate of Zn^2+^ during the electrochemical reaction.

The physical and chemical performance parameters of NVP are shown in Table 1. From the table, it can be seen that the particle size of NVP is relatively fine, and the specific surface area and vibrational density meet the requirements of a cathode material.

**Figure 5:**
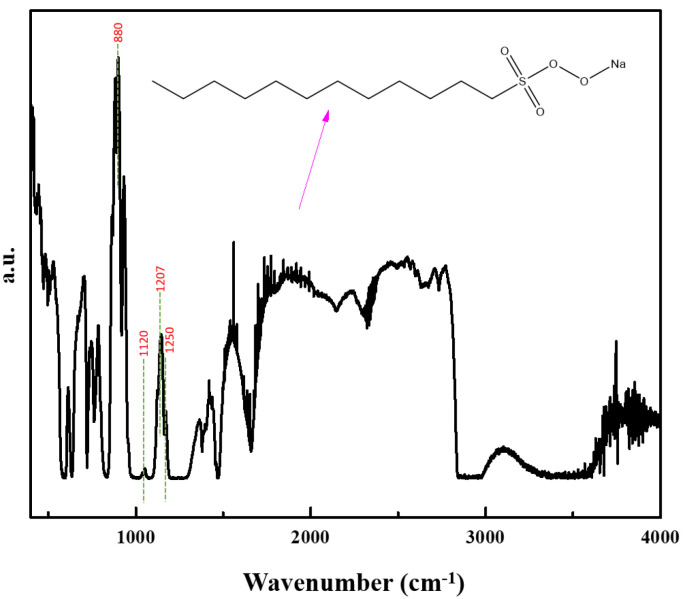
Infrared spectrogram of SDS.

Figure 5 shows the infrared spectra of SDS. As can be seen from the figure, according to the Fourier infrared spectral map of the chemistry manual of the infrared standard library for matching and comparison, we judge that the main component of the SDS sample is sodium dodecyl sulfate, in which the strong absorption peak of the splitting of 1250, 1207 cm^−1^, is caused by the deformation vibration of the -S=O; in addition to the absorption near 1220 cm^−1^, a broad absorption near 1120 cm^−1^ is indicative of sodium alcohol ether sulfate, and the 1120 cm^−1^ absorption band is enhanced as the number of ethylene oxide (EO) additions increases. These peaks have also been mentioned in the other literature.

**Figure 6:**
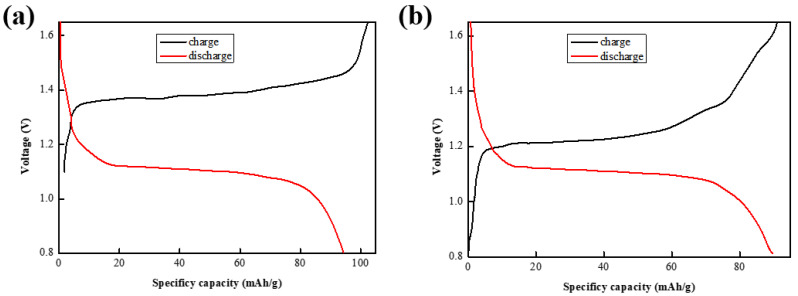
A specific capacity–voltage test plot of (**a**) the first turn of the NVP/Zn cell; (**b**) the second turn of the NVP/Zn cell.

In this paper, sodium vanadium phosphate was used as the cathode material of an aqueous zinc ion battery, Zn flake was used as the cathode, and weakly acidic 1 mol/L ZnSO_4_ was used as the aqueous electrolyte to assemble a 2032 NVP/Zn button type full battery for testing. Figure 6a shows the constant-current charge and discharge curves of the first turn of this battery, from which it can be seen that, during the first charge, two Na^+^ were dislodged from the interior of Na_3_V_2_(PO_4_)_3_ to form the sodium extraction phase NaV_2_(PO_4_)_3_, and a voltage plateau of about 1.40 V was produced during this sodium extraction process; subsequently, during discharge, Zn^2+^ was embedded in the sodium extraction phase to produce another new phase, ZnNaV_2_(PO_4_)_3_, presenting a lower voltage plateau (around 1.25 V).

Figure 6b shows the constant-current charge/discharge curve of the second lap of this cell, where two close voltage plateaus (1.05/1.28 V) can be observed, and it can be surmised that this is the plateau for the reversible embedding/de-embedding of Zn^2+^. Once discharged to 1.2 V, the discharge products of this cell are sequentially Na_3_V_2_(PO_4_)_3_, NaV_2_(PO_4_)_3,_ and ZnxNaV_2_(PO_4_)_3_ (x = 0.25) until the cut-off voltage is equal to 0.6 V. The discharge products of the cell are generally Na_3_V_2_(PO_4_)_3_ and ZnxNaV_2_(PO_4_)_3_, and it can be surmised that this cell is discharged as a process of the Na^+^ and Zn^2+^ co-embedding process [21].

The same results were obtained using non-in situ XRD refinement concerning X-ray absorption fine structure spectroscopy (EXAFS). The zinc storage principle of Na_3_V_2_(PO_4_)_3_ was used to investigate the phase transition of sodium vanadium phosphate materials during battery charge/discharge cycling using several non-in situ methods. Non-in situ XRD and XPS confirmed the charge/discharge process as Zn^2+^/Na^+^ co-embedding/de-embedding.

Based on the reported experimental and computational results, it is known that Na2 is more easily removed from Na_3_V_2_(PO_4_)_3_, and only Na1 exists in NaV_2_(PO_4_)_3_ after Na removal, which means that all Na2 is removed from Na_3_V_2_(PO_4_)_3_ after charging. However, there are still controversies about the specific Na^+^ migration pathway and whether Na1 is involved in the migration process.

**Figure 7:**
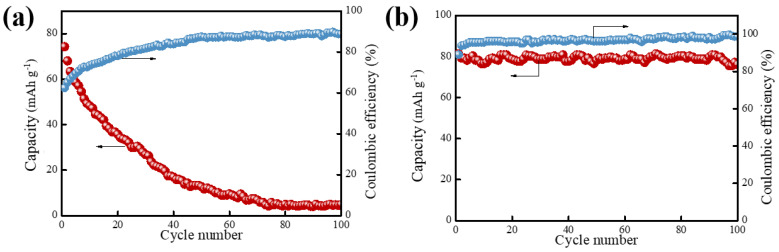
Cycling stability testing of (**a**) NVP/Zn batteries; (**b**) an NVP/Zn battery with SDS addition.

Figure 7a shows the charge/discharge cycle stability test of the NVP/Zn battery at 0.5 C magnification. At a current density of 0.5 C, the capacity reaches 78 mAh/g in the first lap discharge, in which the capacity decay is relatively fast in the first 10 laps, which may be due to the distortion of the lattice of sodium vanadium phosphate caused by the embedding of Zn^2+^, resulting in the loss of the active sites of the cathode material, NVP, and a part of the V is dissolved in the ester electrolyte. The capacity decay slows down in subsequent cycles, decreasing to 4 mAh/g (5% capacity retention) after 100 cycles. This suggests that an increase in the number of cycles corresponds to a rapid decrease in the specific capacity of NVP/Zn during cycling, and therefore it is necessary to improve the cycling stability of NVP/Zn batteries by adding electrolyte additives. The initial Coulombic efficiency of the full cell reaches 58%, which needs some degree of improvement. From the theoretical analysis, it can be concluded that the ionic radius of Zn^2+^ (0.75 Å) is smaller than that of Na^+^ (1.02 Å), so the use of Na_3_V_2_(PO_4_)_3_ for zinc ion batteries is theoretically valuable.

Figure 7b shows the charge/discharge cycles of NVP/Zn batteries at 0.5 C magnification after adding SDS. From the figure, it can be seen that after 100 cycles, the capacity retention rate of the system containing SDS reaches 89%, indicating that the system containing SDS has lower polarization than that of the battery with a pure electrolyte and with an increase in the number of cycles. The initial coulombic efficiency of the full cell is as high as 80%, which can be seen as 12% higher than that of the system without SDS.

SDS, as an additive, is beneficial for reducing polarization during the battery cycling process, delaying the decay of reversible specific capacity, and inhibiting the disappearance of reversible processes, thus improving the cycling stability of the battery. We found that the addition of SDS to the electrolyte system has a significant effect on electrode cycling stability, Coulombic efficiency, and capacity, and the addition of SDS helps to inhibit the side reactions that occur during the cycling process and improve the electrochemical performance of the battery.

**Figure 8:**
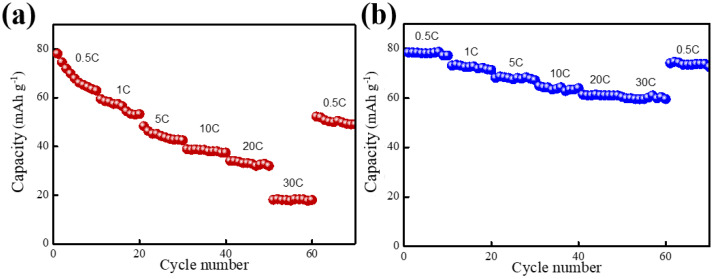
Cyclic voltammetry curves of NVP/Zn cells (**a**) using a reference electrolyte; (**b**) using SDS-containing electrolytes.

The multiplication rate performance of NVP/Zn cells was investigated and the results are shown in Figure 8a. The specific capacity of the NVP/Zn cell decreases rapidly as the multiplication rate increases, and at a high multiplication rate of 30 C, the cell capacity is 19 mAh/g.

From Figure 8b, it can be seen that the multiplicity performance was greatly improved by the addition of SDS, indicating a more rapid electrode reaction process and implying a faster Zn^2+^ diffusion rate. This indicates that the addition of SDS is beneficial to accelerate the transfer process of Zn^2+^. Therefore, the introduction of SDS is beneficial to improve the electrode reaction rate of aqueous zinc ion batteries.

In order to further investigate the electrochemical behavior of NVP/Zn batteries in different electrolytes at room temperature and to analyze the changes in the internal impedance of the batteries during the battery cycling process, EIS tests were performed by cycling the batteries with different electrolytes for 10 revolutions, and the equivalent circuits were fitted using the Zview software to obtain data regarding impedance.

**Figure 9:**
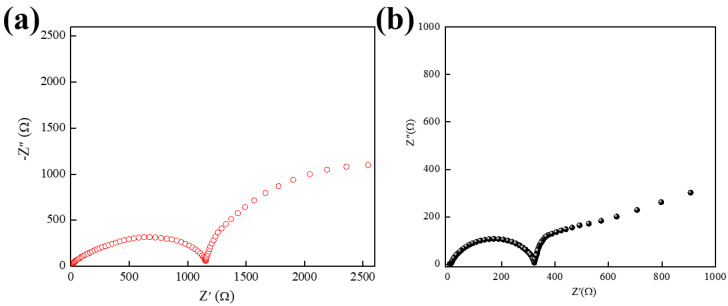
Electrochemical impedance spectra of (**a**) NVP/Zn cells; and (**b**) NVP/Zn cells using SDS-containing electrolyte after cycling.

The electrochemical impedance spectroscopy (EIS) test was performed at the end of the battery discharge and was measured using the constant potential mode; the frequency range used for the test was 0.01 Hz–100 kHz. The EIS test results for the NVP/Zn full cell are shown in Figure 9a. The Nyquist plot for the zinc sulfate electrolyte exhibits two semicircles and shows an increase in both the R_SEI_ and R_CT_ for the cell using the benchmark electrolyte as the number of cycles increases. The impedance R_SEI_ and R_CT_ of the system using the benchmark electrolyte were 75.37 Ω and 96.51 Ω, respectively.

The results of the NVP/Zn cell with SDS added are shown in Figure 9b. The Nyqusit plot of the electrolyte exhibits two semicircles, and the cells using the electrolyte containing SDS have an increase in both their R_SEI_ and R_CT_. The smaller the R_SEI_ value of the cell using the electrolyte containing SDS compared to the cell using the baseline electrolyte indicates that the transport of Zn^2+^ across the interfacial film is accelerated after full activation and that the interfacial film formed by the addition of SDS has a better Zn^2+^ transport rate. The R_SEI_ of the system containing SDS grows slowly at 41.02 Ω. This indicates that the interfacial film of the reference electrolyte is thickened continuously during the cycling process of the battery and the electrodes continue to react with the electrolyte, whereas the addition of SDS forms a more stable, interfacial film, which inhibits the excessive decomposition of the electrolyte. The system containing SDS also has a lower R_CT_ value and a smaller growth rate, with an R_CT_ value of 69.49 Ω and 96.51 Ω for the reference electrolyte system, which shows that the addition of SDS facilitates the migration of Zn^2+^ on the electrode surface and the cell has a smaller polarization level. The above results show that the addition of SDS reduces the impedance during the battery cycling process, reduces battery polarization, improves the diffusion transfer of Zn^2+^, and accelerates the charge transfer process; moreover, the interfacial film generated by the addition of SDS is more stable and accelerates the migration of Zn^2+^ in the interfacial film. The Zn^2+^ can contact the electrode active substance more rapidly and participate in the reaction, which is conducive to improving the electrochemical reaction speed of the zinc electrode.

Zn metal is known to be chemically active and susceptible to chemical reactions when in contact with aqueous electrolytes. The reaction products cover the surface of the Zn metal, and this spontaneously formed interfacial film, if friable, will rupture under the compressive stresses caused by unevenly growing bumps of Zn deposition, resulting in fresh Zn that will continuously react with the electrolyte [22]. In addition, uneven Zn deposition may develop into Zn dendrites, leading to dead Zn or electrode short circuits. Therefore, the uniformity of zinc deposition is crucial for the stability of the zinc metal negative electrode/electrolyte interface, and it is necessary to investigate the effects produced by the addition of SDS on zinc deposition.

**Figure 10:**
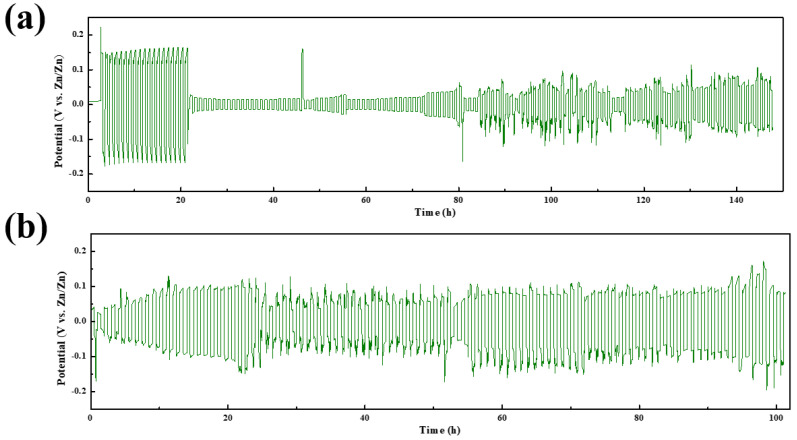
Time−voltage profile of the (**a**) Zn/Zn symmetric cell; and (**b**) the Zn/Zn symmetric cell after addition of SDS.

The deposition process of the metal anode in zinc ion batteries is a major challenge in batteries because uneven deposition not only causes low Coulombic efficiency but also leads to battery failure. The regulation of the zinc deposition process mainly includes inhibiting the growth of zinc dendrites and influencing the texture growth. The regulation of dendrite growth is most intuitive in the zinc deposition process. Texture changes are not easy to see visually, which represents the crystalline surface of the zinc metal in contact with the electrolyte, but indirectly affects zinc anode corrosion, dendrite growth, and the performance of other anodes. As shown in Figure 10a, a Zn/Zn symmetric cell was assembled using the reference electrolyte, and this symmetric cell was tested with constant current charge and discharge.

The electrochemical reaction at the zinc electrode in the negative electrode proceeds via a dissolution–precipitation reaction. The reaction mechanism involves the formation of a supersaturated zincate solution through the following electron transfer reaction:

Zn + 4OH^−^ ⇔ Zn(OH)_4_^2−^ + 2e^−^ (1)

Zn (OH)_4_^2−^ forms porous zinc oxide on the electrode surface through the following chemical reaction:

Zn(OH)_4_^2−^ ⇔ ZnO + 2OH^−^ + H_2_O (2)

Inhibiting the growth of zinc dendrites on the negative electrode surface of aqueous zinc ion batteries can be achieved by using electrostatic shielding mechanisms and increasing the nucleation rate to make the zinc grains on the negative electrode surface more homogeneous in order to increase the overpotential for zinc deposition and to limit the two-dimensional diffusion of Zn^2+^ on the anode surface. Increasing the overpotential of aqueous zinc ion batteries helps to inhibit Zn^2+^ deposition, and if the overpotential is more appropriate, it essentially does not interfere with deposition but inhibits the growth of dendrites.

The change of overpotential affects the nucleation quantity parameter, and according to the deposition nucleation mechanism, increasing the deposition overpotential of Zn^2+^ easily causes an increase in the nucleation quantity and speed of the negative metal surface, which inhibits the growth of dendritic crystals. At the same time, the lower amount of nucleation on the surface of zinc metal caused the excessive deposition of Zn^2+^ somewhere on the surface of the negative electrode, which causes uneven zinc growth and further causes the generation of dendritic crystals. Some additives can improve the overpotential of zinc deposition, mainly surfactants, metals, ionic liquids, etc. [23].

To further investigate the effect of SDS on zinc deposition when it is used as an additive, a Zn/Zn symmetric cell was assembled using the electrolyte containing SDS, and the symmetric cell was subjected to a constant-current charge/discharge test, the results of which are shown in Figure 10b. We can find that the addition of SDS effectively reduces the overpotential during Zn deposition, and the overpotential is kept at a low level overall. Local amplification of the cycling process of 10~13 h shows that the addition of SDS reduces the nucleation overpotential and the overpotential after the generation of concentration polarization, indicating a smoother zinc deposition process. The cell using the reference electrolyte shows a process of sudden decrease in overpotential at 22 h. This process was delayed to 100 h after the use of SDS, indicating that the addition of SDS makes the deposition of Zn^2+^ more stable. The cell using the reference electrolyte shows a rapid increase in overpotential with a sudden drop after 48 h, representing the process of partial zinc dendrite growth from rapid growth to detachment. The initial nucleation overpotential, which was higher at the beginning of the cycle, basically disappears during the subsequent cycle [24]. At this point, the deposition rate of Zn^2+^ in symmetric cells is mainly controlled by the diffusion process, and the thicker layer of dendrites restricts the diffusion rate of Zn^2+^, thus requiring more time to reach the deposition site. In contrast, in the cell using SDS as an additive, there is no obvious process of rapid potential increase and sudden drop, and the nucleation overpotential existed for a longer time, indicating a more uniform zinc deposition process. The above phenomena indicate that the addition of SDS makes the deposition of Zn^2+^ on the surface of zinc flakes more uniform, and the diffusion and deposition process of Zn^2+^ is subject to less resistance. Compared to a specific cycling process, the cell using SDS has a lower initial nucleation overpotential, which indicates that Zn^2+^ has less resistance when diffusing to the surface of the negative electrode of zinc metal for deposition, and the deposition process is more rapid and stable.

Molecular orbital energy is commonly used to predict the oxidation and reduction trends of organic molecules. Theoretically, the larger the HOMO value, the easier it is to lose electrons for an oxidation reaction, and the smaller the LUMO value, the easier it is to gain electrons for a reduction reaction. Therefore, the orbital energy can be used as a reference for redox stability. After the addition of SDS, the stability of the electrolyte is improved, which may be because the actual redox potential will be affected by the solution environment and solvation structure; SDS is less affected by the solution environment, and thus the redox potential is relatively stable [25].

**Figure 11:**
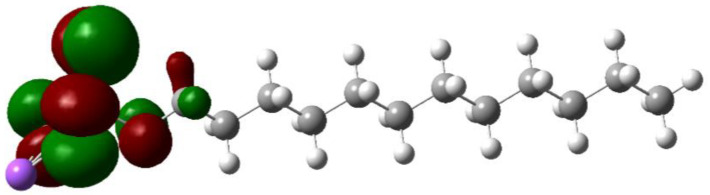
HOMO/LUMO diagrams for SDS.

The HOMO and LOMO values of SDS were calculated using Gaussian software, and Figure 11 shows the comparison of the HOMO and LUMO energies of SDS.

Table 2 shows the orbital energy and physical property parameters of SDS, and Table 3 shows the optimized coordinate parameters of SDS. It can be seen that the addition of SDS can make the electrolyte redox stability improve. The formation of the SEI film is mainly related to the basic properties of electrolyte additives, the most important of which are the HOMO and LUMO energy level properties of additives. For example, electrolyte decomposition and side reactions can easily occur at the electrode/electrolyte interface under the extremely harsh conditions of high temperature and high pressure. According to the molecular orbital theory, the oxidation potential of a molecule is related to the HOMO orbitals, and the reduction potential of a molecule is related to the LUMO orbitals.

Thus, if the LUMO orbital of the selected electrolyte additive is lower than the anodic chemical potential, the electrolyte additive is likely to reductively decompose on the anodic surface to form a stable SEI film which protects the anode from further electrolyte decomposition. Similarly, if the HOMO energy level of the selected additive is higher than the cathodic chemical potential, the additive is likely to deposit a cathodic electrolyte interface (CEI) on the cathode to prevent the further decomposition of the electrolyte. Thus, the formation of an interfacial film reduces the endless consumption of electrolytes and mitigates the build-up of by-products. In addition, cathodic dissolution and anodic corrosion, which are particularly severe in the case of hyperactivity, are suppressed due to the separation from the electrolyte and the protection of the interfacial film.

To verify the modulation of the coordination state of Zn^2+^ by SDS, DFT theoretical calculations have been carried out for the electrolyte system of the battery, and the interaction of the two systems, Zn-H_2_O and Zn-SDS, can be calculated and compared on this basis. It can be seen that the interaction energy of Zn with SDS is −0.19 eV, and this value can be seen to be much lower than that of Zn with water, which is −0.05 eV. The lower interaction energy of Zn with SDS suggests that in aqueous solutions, Zn prefers to coordinate with SDS.

Compared with the Zn-H_2_O system, the electronic interaction between Zn and SDS is stronger, and thus the electron cloud density around Zn is higher in this system. The above DFT results theoretically demonstrate that SDS can achieve the modulation of the coordination state of Zn^2+^.

Based on the above discussion, it was found that the SDS anion preferentially adsorbs on the (002) surface of the zinc anode, forming a protective layer that prevents the adsorption of free water molecules and modifies the electrode/electrolyte interface. The SDS additive greatly inhibits the contact of water molecules with the interface, reduces the generation of by-products, and effectively prevents the growth of dendrites. Therefore, the cycle life of Zn/Zn symmetric cells can be extended with the help of SDS additives.

In summary, increasing the voltage window of aqueous ZnSO_4_ batteries can be performed through two methods, including reducing the HOMO level of water (i.e., increasing the OER potential) and increasing the LUMO level of water (i.e., decreasing the HER potential). A typical example of increasing the voltage window is the addition of SDS additives to a 1 M ZnSO_4_ aqueous electrolyte, which leads to an electrochemical stability window of 2.5 V for aqueous ZIBs [26]. The SDS additive forms a hydrophobic layer at the electrode/electrolyte interface. When water passes through this hydrophobic layer, a high energy barrier is created, and thus water is not easily cracked to produce oxygen, thus enlarging the electrochemical stability window. In addition, this dehydration mechanism hinders the contact between water and the electrode on the one hand, thus improving the electrochemical stability of water. On the other hand, it facilitates the desolvation of Zn^2+^, thus preventing dendrite growth and by-product generation and improving Coulombic efficiency [26].

The reaction for the formation of zinc oxide on the negative zinc metal surface is a dissolution–precipitation process. During discharge, the conversion of zinc powder to zinc oxide occurs in two steps: First, the zinc on the anode reacts with OH^−^ to form Zn(OH)_4_^2−^, the so-called “dissolution process”. Then, the zinc acid radical ions are converted to zinc oxide, the so-called “deposition process” or “precipitation process”. The first step is an electrochemical reaction and therefore involves electron transfer, which occurs only at the conducting sites of the fresh zinc. The rate of the electrochemical reaction therefore depends on the diffusion of ions and the effective surface area of the zinc. The second step is a chemical reaction, the rate of which depends on the zincate ion. The solubility of the zincate ion determines the rate of reaction.

During the discharge process, when the concentration of anionic SDS surfactant is particularly low, Zn^2+^ dissolves slowly from each zinc crystal. As a result, the accompanying depletion of OH^−^ within the anode gel may be offset by the diffusion of OH^−^. In addition, the subsequent reaction of Zn(OH)_4_^2−^ with ZnO occurs slowly due to the low supersaturation of Zn(OH)_4_^2−^ in the vicinity of each parent zinc crystal. Due to the slow deposition rate, the newly formed ZnO tends to preferentially participate in the already deposited ZnO crystals, i.e., the growth rate is less than the nucleation rate.

Therefore, in the presence of anionic SDS surfactant, a fine ZnO layer is formed on the anode surface. In the presence of a high concentration of the surfactant, the ZnO crystal growth is regular with less of a needle-like morphology, which affects the penetration of the electrolyte into the micropores and ultimately reduces the proton mobility on the zinc surface and restricts the diffusion of ions. This phenomenon affects the discharge performance of the battery and gradually improves the total lifetime of the battery. In addition, the passive films formed on the zinc electrodes of the battery have active properties, i.e., the anionic SDS surfactants reduce the dissolution rate, especially when they consist of semiconducting films of fine ZnO crystals.

Since the performance of zinc ion batteries involves the influence of the water/zinc interface, it is necessary to investigate the effect of the interface on the electrochemical performance of aqueous batteries. It can be seen that in aqueous zinc ion batteries, the electrolyte additive SDS, which is generally subject to surface tension, can be spontaneously distributed at the interface between the electrode and the electrolyte during the charging and discharging cycles of the battery, hindering direct contact between the active water and Zn. While better inhibiting the occurrence of side reactions, it can also rebuild the interfacial environment to change the deposition kinetics of Zn^2+^, thus inhibiting the growth of Zn dendrites. The interfacial layer can directly prevent direct contact between the electrolyte and the Zn negative electrode or can reduce the number of water molecules reaching the surface of the Zn negative electrode through desolvation to mitigate side reactions. In addition to surface coatings, electrolyte additives can also inhibit the growth of zinc dendrites. Zinc dendrite growth can be inhibited by an electrostatic shielding layer, which increases the nucleation rate and limits the two-dimensional diffusion mechanism, thereby significantly improving battery performance. According to previous studies, electrolyte additives can be divided into ionic and non-ionic additives. It is worth noting that the SDS selected in this paper has significant advantages in terms of price and safety over other additives that have been reported in zinc ion batteries, showing greater potential for market application, as shown in Table 4.

SDS ionic additives can further improve the deposition sites of Zn^2+^ by electrostatic shielding or the ion adsorption mechanism, inhibit the growth of zinc dendrites, and obtain a more uniform zinc deposition layer. A more stable electrolyte/zinc anode interface can be obtained by using ionic additives. SDS ionic additives, which have the properties of inhibiting anodic dissolution and forming a shielding layer on the surface of the zinc negative electrode, could be a priority for the development of electrolyte additives. The polar molecules of SDS can preferentially reach the tip of the initial nucleating particles to form an electrostatic shielding layer, which prevents further deposition of Zn^2+^ and thus inhibits the growth of zinc dendrites [26].

Usually, the inhibition effect of electrolyte additives on dendrites as well as side reactions in aqueous zinc ion batteries is concentration-dependent. To investigate the effect of SDS concentration on zinc-negative electrodes, the ionic conductivity and the corresponding corrosion inhibition were explored for different concentrations of SDS solutions. Generally, as the concentration of SDS additives in the solution increased, the ionic conductivity of the electrolyte solution for aqueous zinc ion batteries showed a continuous decrease. The corrosion inhibition of SDS in zinc battery systems is an important aspect of battery performance optimization. To investigate this process, the effect of different concentrations of SDS on the corrosion behavior of zinc anodes was systematically assessed by performing Tafel curve tests. Tafel curves can provide key data on corrosion potential (E_corr_) and corrosion current (i_corr_), which reflect the stability and corrosion rate of a material in a corrosive environment. It was found that the corrosion potential of zinc shifted in a more positive direction and the corrosion current decreased as the concentration of SDS increased. This indicates that SDS, as a buffer and possible complexing agent, can effectively enhance the chemical stability of the zinc anode and slow down its corrosion process. A more correct corrosion potential indicates an increase in the thermodynamic stability of zinc, while a lower corrosion current implies a decrease in the corrosion rate of zinc.

**Figure 12:**
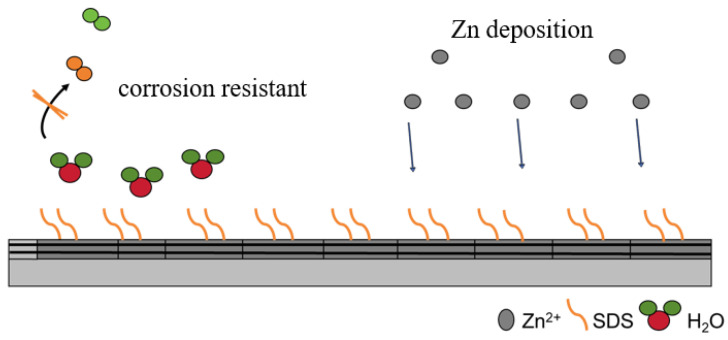
Corrosion inhibition mechanism diagram of SDS additives.

The decision to choose 1% SDS for the study was based primarily on the desire to balance ionic conductivity and corrosion rate. SDS may not only act as a surfactant to reduce zinc corrosion but may also influence the behavior of Zn^2+^ in the electrolyte through the formation of stable complexes. During zinc deposition and stripping, complexation may assist in the formation of a more homogeneous deposition layer and a more controllable stripping behavior, thereby improving the cycling stability and efficiency of the cell. Notably, it would be of great interest to conduct further experiments to test this hypothesis, such as examining the deposition morphology, electrochemical impedance, and long-term cycling performance of zinc at different SDS concentrations. These experiments will help form a deeper understanding of the mechanism of action of SDS in zinc batteries and how to optimize its concentration and conditions of use to achieve the best battery performance. As shown in Figure 12, with the increase in SDS concentration in the battery electrolyte, Zn showed more positive corrosion potential and a smaller corrosion current, a phenomenon that indicates that the addition of SDS can effectively inhibit the corrosion of the Zn negative electrode. Considering the different variations of ionic conductivity and a corrosion rate with the concentration of SDS, we chose a 1% concentration of SDS to study the role played by the complexing agent in zinc deposition stripping.

## 3. Experimental Section

### 3.1. Preparation of Electrolyte

A 1 mol/L zinc sulfate electrolyte was prepared in a constant volume flask using zinc sulfate and deionized water. The configured ZnSO_4_ electrolyte was used as the base electrolyte, and SDS was weighed and added to it per 1% mass of zinc sulfate to obtain a new electrolyte formulation. It is denoted as STD + 1% SDS, indicating that a 1% mass fraction of SDS was added to the base electrolyte.

### 3.2. Electrochemical Measurements

A CR2032 button type NVP/Zn battery was assembled to test its cycling performance and multiplication performance. Sodium vanadium phosphate (NVP), synthesized using the hydrothermal method, conductive agent Super P, and polyvinylidene fluoride (PVDF), was mixed according to the mass ratio of 8:1:1 to prepare the cathode of the NVP/Zn full battery. The obtained cathode-mixed material slurry was uniformly coated on carbon-coated aluminum foil using the scraping method. After pre-drying at 50 °C for 3 h, the material was transferred to a vacuum drying oven at 60 °C for 12 h to be used as the cathode of the NVP/Zn all-cell battery, and was then assembled into a CR2032 button cell. The electrochemical properties of the assembled cells were tested in the Blue Battery Test System.

Zn/Zn symmetric cells were assembled and subjected to constant-current charge/discharge tests to evaluate the Zn shedding and deposition behavior during cell cycling. A reference electrolyte/electrolyte containing SDS was injected into the cell. The Zn/Zn symmetric battery was subjected to constant-current charge/discharge testing using the Blue Power Battery Test System (CT3001A 1U) to gain insight into the behavior of zinc during cycling and to assess the stability of the zinc negative electrode and dendrite formation.

Electrochemical impedance spectroscopy (EIS) tests were performed on NVP/Zn batteries cycled for 10 cycles using a reference electrolyte as well as an electrolyte containing SDS, the equivalent circuits were fitted, and data on impedance were obtained using Zview software. The electrochemical behavior of NVP/Zn batteries in different electrolytes at room temperature was evaluated and the changes in the internal impedance of the batteries during cycling were analyzed.

### 3.3. Characterization

The battery was disassembled after 100 cycles, and the NVP-positive electrode was removed for cleaning treatment and dried for subsequent testing. A FLEX SEM 1000 scanning electron microscope was used to observe the morphology of the surface of the NVP positive electrode of the NVP/Zn battery after cycling. Energy dispersive X-ray spectrometry (EDS) was used to analyze the elemental distribution on the surface of the NVP anode and combined with thermogravimetric analysis to do an elemental ratio analysis. The functional group structure of SDS, an electrolyte additive for aqueous zinc ion batteries, was analyzed by Fourier Transform Infrared Spectroscopy (FTIR).

### 3.4. Calculation Section

The Highest Occupied Molecular Orbital (HOMO) and Lowest Unoccupied Molecular Orbital (LUMO) values of SDS were calculated using the Gaussain software to analyze the effect of SDS addition on the redox stability of the electrolyte. Density-functional theory (DFT) calculations were carried out on the electrolyte system of the battery to verify the regulation of the coordination state of Zn^2+^ by SDS.

## 4. Conclusions

In summary, sodium dodecyl sulfate was introduced as an electrolyte additive for aqueous zinc ion batteries. Through cyclic charge/discharge tests, it can be concluded that adding SDS is beneficial for improving the discharge-specific capacity of NVP/Zn batteries at a high voltage, and the capacity retention is enhanced. Using a battery containing 1% SDS, a discharge-specific capacity of 71 mAh/g was still achieved after 100 cycles of charging at a current density of 1 C, and the capacity retention rate was as high as 89%. The EIS results further showed that the addition of SDS resulted in a significant reduction in the charge transfer impedance of the NVP/Zn battery during charge/discharge cycling, reducing the polarization of the battery. As a result of these advantages, the cycling stability of NVP/Zn batteries during charge/discharge processes was substantially enhanced. The modulation of zinc deposition on the surface of the Zn negative electrode through the addition of SDS was also explored. SDS adsorbed on the anode surface to form a protective layer through electrostatic action, which inhibited the contact of free water molecules with the interface and effectively suppressed the growth of dendritic protruding dendrites. With the help of SDS additives, the cycle life of Zn/Zn symmetric batteries can be extended. It is concluded that SDS has the function of regulating Zn^2+^ deposition and can be introduced as an electrolyte additive for zinc ion batteries, which can improve the stability of aqueous Zn-based batteries.

## Figures and Tables

**Table 1:** Physical and chemical properties of NVP.

Main Parameters				
Test Items			Unit	Measured Value
Chemical properties	Chemical formula		/	Na_3_V_2_(PO_4_)_3_
Physical properties	Particle size distribution	D10	µm	6.2
		D50	µm	18
		D90	µm	32
	Appearance		/	Black powder
	Specific area		m^2^/g	≤178
	Tap density		g/cm^3^	5.6

**Table 2:** Orbital energies and physical parameters of SDS.

	HOMO/eV	LUMO/eV	Physical Parameters		
			Melting Point/°C	Stickiness/cP (20 °C)	Dielectric Constant
SDS	−0.07651	−0.26734	206	19~31	0.008

**Table 3:** Optimized coordinate parameters for SDS.

Element	X-Coordinate	Y-Coordinate	Z-Coordinate
C	3.25303700	−0.39756200	0.22237300
C	1.99748000	0.39111200	−0.13814500
C	0.71381500	−0.38406200	0.22165700
C	−0.56924300	0.39146100	−0.12872500
C	−1.85695900	−0.37970900	0.21331900
C	−3.14258900	0.39337300	−0.13255000
C	−4.43034900	−0.38123100	0.20171500
C	−5.71681100	0.39191300	−0.14074100
C	−7.00446800	−0.38458200	0.18962700
C	−8.29118300	0.38923700	−0.14978400
C	−9.57943300	−0.38752000	0.17902600
C	−10.85880000	0.39408500	−0.16022100
O	4.39417100	0.45572400	−0.18344000
S	5.99821500	−0.39318500	−0.16374800
O	6.68650300	−0.06487300	1.32634700
O	6.88913400	0.51261700	−1.24131500
O	5.74017700	−1.95582400	−0.51035300
Na	8.17510500	1.32509400	0.39728100
H	3.30632400	−1.34730300	−0.31802600
H	3.31484300	−0.59042900	1.29950200
H	2.01546200	0.61109100	−1.21280300
H	2.01329300	1.35419900	0.38767600
H	0.71368200	−0.61782300	1.29693800
H	0.70977600	−1.34940500	−0.30588500
H	−0.56466600	0.63332500	−1.20233500
H	−0.56857400	1.35425400	0.40448500
H	−1.85909800	−0.62563700	1.28641000
H	−1.85670900	−1.34125400	−0.32246800
H	−3.13801600	0.64383500	−1.20459100
H	−3.14553800	1.35288300	0.40717900
H	−4.43374800	−0.63444300	1.27318100
H	−4.42792400	−1.33946800	−0.34020300
H	−5.71221400	0.64754800	−1.21161700
H	−5.72057400	1.34898300	0.40336100
H	−7.00804900	−0.64232300	1.26006000
H	−7.00147900	−1.34063500	−0.35626300
H	−8.28831000	0.64815900	−1.22000700
H	−8.29572600	1.34492400	0.39697900
H	−9.58081500	−0.64797500	1.24788600
H	−9.57543800	−1.34139500	−0.36892900
H	−11.75806500	−0.18284300	0.08523600
H	−10.89938600	0.63872000	−1.22922900
H	−10.90350300	1.33771100	0.39814300

**Table 4:** A comparison of the price and safety of electrolyte additives used in zinc ion batteries.

Additives	Price	Safety	References
Ethyl ether (Et_2_O)	Expensive	Toxic	[16]
Hexadecyl trimethyl ammonium Bromide (CTAB)	Cheap	Toxic	[18]
Diethyl ether	Cheap	Toxic	[27]
Graphene oxide	Expensive	Harmful	[28]
N, N-dimethyl acetamide (DMA)	Expensive	Harmful	[29]
SDS	Cheap	Harmful	This work

## Data Availability

No new data were created or analyzed in this study. Data sharing is not applicable to this article.
